# Investigation of proteinaceous paint layers, composed of egg yolk and lead white, exposed to fire-related effects

**DOI:** 10.1038/s41598-020-75876-y

**Published:** 2020-11-03

**Authors:** Lea Legan, Klara Retko, Kelly Peeters, Friderik Knez, Polonca Ropret

**Affiliations:** 1 Research Institute, Conservation Centre, Institute for the Protection of the Cultural Heritage of Slovenia, Poljanska 40, 1000 Ljubljana, Slovenia; 2 InnoRenew CoE, Livade 6, 6310 Izola, Slovenia; 3 University of Primorska, Andrej Marušič Institute, Muzejski trg 2, 6000 Koper, Slovenia; 4 Slovenian National Building and Civil Engineering Institute, Dimičeva 12, 1000 Ljubljana, Slovenia; 5 Museum Conservation Institute, Smithsonian Institution, 4210 Silver Hill Rd., Suitland, MD 20746 USA

**Keywords:** Infrared spectroscopy, Characterization and analytical techniques, Mass spectrometry, Environmental sciences, Natural hazards, Materials science, Chemistry, Analytical chemistry

## Abstract

Fires can have a negative impact on the environment, human health, property and ultimately also on various objects of cultural heritage (CH). This paper deals with an investigation into the degradation of selected proteinaceous paint layers that were exposed to fire-related effects (i.e., fire effluents and/or high temperatures) in a modified cone-calorimeter system. Paint layers of egg yolk adhesive (E) and lead white tempera (E + LW) were exposed to fire-related impacts on top of a CH stack and in a specially designed CH test chamber. On the CH stack, the proteinaceous paint layers were exposed to fire effluents and high temperatures, while in the CH test chamber, the samples were exposed mainly to fire effluents. The molecular changes to the exposed paint layers were analysed by invasive and non-invasive spectroscopic analyses (i.e., FTIR and Raman spectroscopy) and complimented with pyrolysis-GC–MS, while the colour changes were evaluated using colourimetry. It was concluded that the proteinaceous binder degrades into aromatic amino acids and/or fatty acids after exposure to the overall impacts of the fire. Aromatic amino acids were detected by means of the FTIR and py-GC–MS analyses. In the case of the lead white tempera exposure, partial dissociation of the lead white pigment was confirmed by the detection of alteration products, such as lead oxide and lead carbonate. Moreover, the investigation of the E + LW samples exposed for longer times revealed the presence of lead carboxylates. On the other hand, no significant molecular changes were observed with the CH samples exposed to fire effluents in the CH test chamber. The research offered us an insight into the fire-induced effects on selected paints for the first time.

## Introduction

Fires are one of the greatest threats to the environment and pose a great risk to cultural heritage (CH) assets. Their effects are almost always disastrous and no matter how extensive the fire is, the damage is usually very large and irreversible. Unfortunately, there are already many examples of world-famous CH and/or historical sites and objects, such as Notre Dame Cathedral in Paris, St. Sava Serbian Orthodox Church in New York, the historic ship Cutty Sark, Kapellbrücke bridge in Switzerland, Kasubi Tombs in Uganda, and historic buildings in Kosovo and BiH, that were destroyed by fire or intentional arson attacks^1^. In addition to the physical damage (i.e., destroying infrastructure, vegetation, etc.), which is mainly caused by the heat released during combustion, fires can also cause atmospheric pollution and water contamination due to the emission of fire effluents during the burning^2^. In recent years many guidelines and fire-safety engineering strategies were presented for the protection of tangible and intangible cultural heritage against fire^3^. However, the provision of fire safety, as well as appropriate and careful post-fire conservation treatments, remains ambiguous in the field of CH^3,4^.

The cone calorimeter is one of the most reliable bench-scale devices for testing the behaviour of fires and the effects on the environment, as well as the various health and safety issues related to fire^5^. This instrument-based method makes possible the monitoring of various properties of the fire in a specific fire scenario, such as the ignition time, critical heat flux, smoke production, and flame spread^6,7^. In the field of CH, several studies have been published regarding the combustion characteristics of different historical building materials and their fire-retardant coatings. Recently, Chorlton et al. investigated the fire-related performance of historic timber and contemporary encapsulations as well as the progression of historic encapsulations used for fire over time^4,8^. Using a cone calorimeter, Zhou et al. examined the effects of weathering and fire retardants on the reaction-to-fire performance of a cedar tree^9^. Additionally, the same instrumentation was used to determine the different parameters (i.e., heat release rate, concentration of CO and CO_2_, etc.) in order to investigate the feasibility and efficiency of water mist suppressing the fire, which occurred in Buddhist temples in North China^10^. Cone-calorimeter experiments were also carried out by Su et al. to determine the characteristics of combustible materials in historical Japanese-style wooden buildings^11^. But, to the best of our knowledge, the cone calorimeter has never been used as a device for the exposure of CH model samples to controlled burning conditions or for an investigation into the degradation of paint layers.

Egg tempera is one of the traditional proteinaceous binding media and consists of egg yolk and/or egg white. Egg yolk is a complex system and consists of water, proteins, carbohydrates and lipids. Lipids, including triglycerides, phospholipids and sterols (i.e., cholesterol) are present in the highest proportions in egg yolk (about 65% of the dry matter)^12,13^. Egg yolk proteins are mainly comprised of phosvitin, livetin, low density (LDL) and high density (HDL) lipoproteins^14,15^. If egg and egg tempera paints are exposed to high temperatures, denaturation processes begin in the protein part of egg yolk and/or egg white. Thermal denaturation of the egg proteins initially alters their quaternary and tertiary structure, but with increased time and/or temperature, secondary structures (i.e., α-helices and ß-sheets) can also unravel. The primary structure is usually only affected by extreme heat. However, thermal denaturation is very often irreversible, as with the disturbances of the secondary structures, inter- or intramolecular bonds can be established between the peptides’ amino acid residues (“crosslinking”) of the types and in the positions that do not occur in their native (biological) states. Furthermore, degradation to shorter poly- or oligopeptides can occur. As a consequence, the protein loses several characteristics, including its biological activity and water solubility. Amino acids are very stable molecules with high melting points^16^. Olafsson et al. showed that tyrosine (Tyr), from among all 20 α-amino acids, has the highest melting point (Tm =  ~ 316 °C), whereas L-glutamine (Gln) (Tm =  ~ 196 °C) and L-glutamic acid (Glu) (Tm =  ~ 210 °C) have the lowest melting points^16^. On the other hand, high temperatures also affect the lipidic part of the egg tempera (i.e., egg yolk). The accelerated degradation of lipids consists of various chemical processes, leading to bond cleavage, oxidation and cross-linking reactions, the hydrolysis of the ester bonds, the formation of free fatty acids, and in the presence of a metal cation, the formation of metal soaps^13,17^.

The degradation of CH materials can be assessed using several analytical techniques, with spectroscopic techniques being the most commonly used. It was already shown that Raman spectroscopy, infrared spectroscopy and GC–MS can offer detailed information for material characterisation as well as about the degradation and degradation products^18^. Furthermore, colourimetry can support an investigation on the basis of an assessment of the colour change.

This paper is devoted to an investigation of proteinaceous paint layers that were exposed to a well-defined fire scenario by means of a modified cone calorimeter. To better understand the overall impact (i.e., thermal effects and the effects of fire effluents (FEs)) of the fire, two sets of egg tempera model samples were placed in special test chambers (where the impact of fire effluents dominated) and in a specially designed steel mesh holder mounted on top of the cone calorimeter stack (exposure to fire effluents and high temperatures). CH model samples were exposed on top of the stack for different time periods. Molecular changes to the exposed CH samples were determined using different spectroscopic techniques (non-invasive and conventional FTIR spectroscopy, Raman spectroscopy). Furthermore, pyrolysis GC–MS was performed on selected model samples in order to validate the results collected using vibrational spectroscopies, while the colour change was evaluated with colorimetry.

## Materials and methods

### Cultural heritage model samples

Two sets of proteinaceous model samples (see Table 1) were prepared according to traditional art technology^19,20^. In the first set an egg yolk adhesive (E) was prepared from free-range hens’ eggs. The egg yolks were separated and then stirred and diluted with tap water. Then, 3% acetic acid was added as a preservative. For the second set (E + LW), the pigment lead white (LW) (Cremnitz White (46,000), Kremer Pigmente GmbH & Co. KG) was ground with the proteinaceous binder (egg yolk adhesive) from the first set. The binder/pigment ratio (w/w) was 70/30. The prepared paints were applied to glass microscope slides with a brush and served as the model samples. The CH model samples were exposed to fire-related effects at two different locations. The first location was on top of the CH stack, where the CH model samples of dimensions 13 mm × 76 mm were exposed to high temperatures and fire effluents for 30 s, 1 min, 5 min and 20 min. The second location for the exposure of the CH model samples of dimensions 26 mm × 76 mm to fire effluents was in a special CH test chamber.

**Table 1:** Description of the samples exposed to fire-related effects (FE).

Sample description	Sample symbol			Location of exposure to FE	Time of exposure to FE
	Batch 1	Batch 2	Batch 3		
Egg yolk adhesive	E-2a	E-18a	E-22a	CH stack	30 s
	E-2b	E-18b	E-22b	CH stack	1 min
	E-3b	E-19a	E-23a	CH stack	5 min
	E-3a	E-19b	E-23b	CH stack	20 min
	E-4	E-20	E-24	CH test chamber	20 min
	E-5	E-21	E-25	CH test chamber	20 min
Lead white tempera	E + LW-13a	E + LW-31a	E + LW-35a	CH stack	30 s
	E + LW-13b	E + LW-31b	E + LW-35b	CH stack	1 min
	E + LW-14a	E + LW-32a	E + LW-36a	CH stack	5 min
	E + LW-14b	E + LW-32b	E + LW-36b	CH stack	20 min
	E + LW-23	E + LW-33	E + LW-37	CH test chamber	20 min
	E + LW-24	E + LW-34	E + LW-38	CH test chamber	20 min

### Burning material

During the experiment, untreated, cut-to-size spruce (*Picea abies*) blocks were burnt in the cone calorimeter. The size of the blocks was 99 mm × 99 mm, and 20 mm thick. The density of the spruce varied between the individual test runs. The mean value of the density was 488 kg/m^3^, with a standard deviation of 68 kg/m^3^. The moisture content of the spruce blocks was 13.4% wt, with a standard deviation of 0.9% wt.

### Instrumentation

#### Cone calorimeter

**Figure 1:**
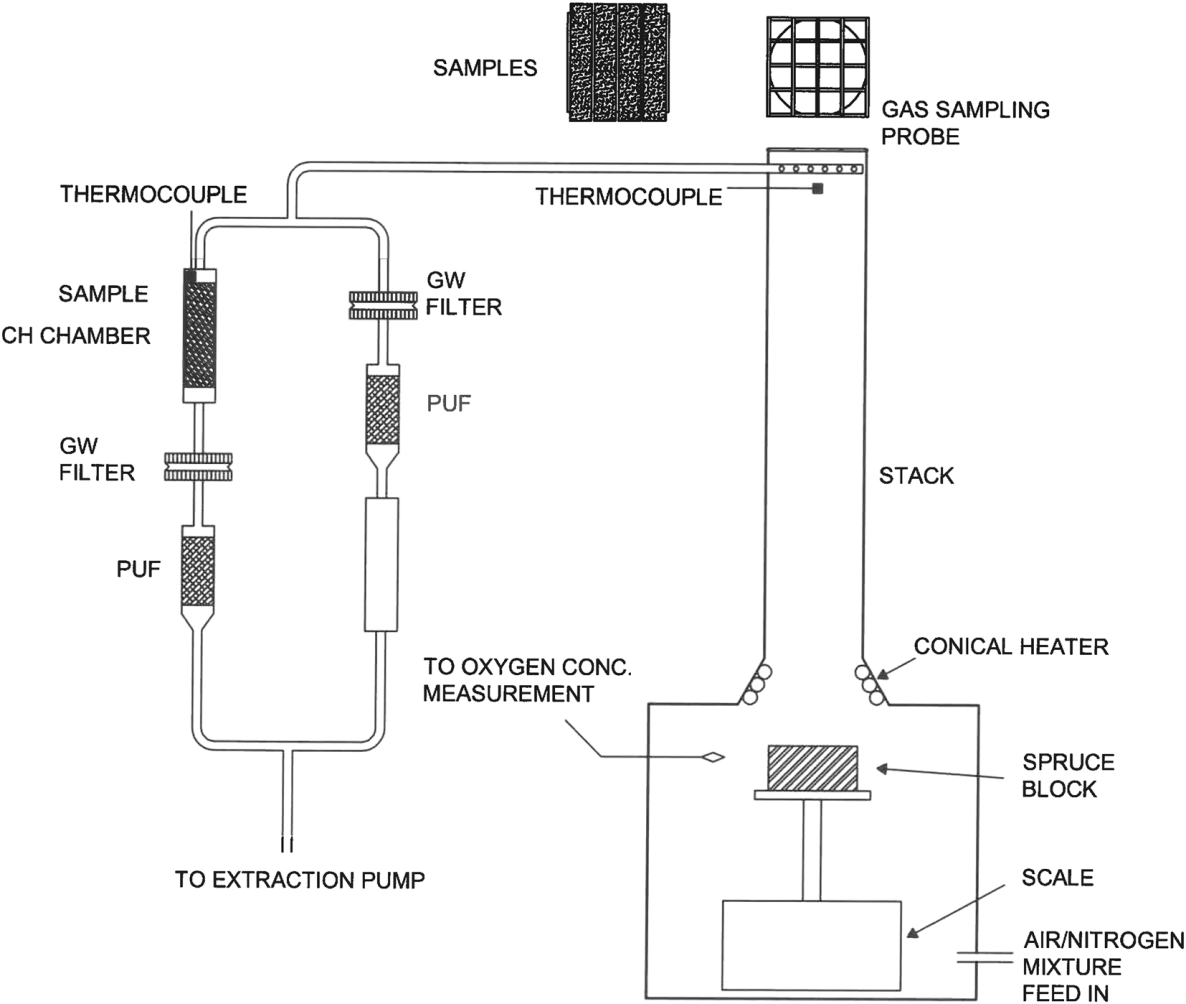
Experimental setup: at the top of the stack the sample holder is attached; the two sampling paths include a chamber for holding the sample, a holder and a glass-wool disc and holder for the PUF adsorption foam.

The spruce blocks were burnt in a modified, controlled-atmosphere, cone calorimeter (modified CACC) (Fig. 1). The test rig is based on a standard cone calorimeter (ISO 5660-1), enclosed in a tight chamber, with a separate feed of air/nitrogen mixture and an opening for the exhaust at the top. The volume of the chamber is 35 dm^3^. The conical heater is a standard one, as described in ISO 5660-1, with a bottom diameter of 160 mm, capable of producing a heat flux impinging on the surface of the sample with an intensity of up to 100 kW/m^2^. On top of the opening, a stack of 100-mm inner diameter and 500-mm length was added. The bottom of the stack was shaped in such a way that it forms a flange with the top of the CACC, which provides a tight fit of the two pieces.

The modified CACC chamber was connected to an air/nitrogen mixing station to control the concentration of oxygen in the mixture, supplied to the modified CACC chamber. The feed-in flow to the chamber was 2.4 l/s (1/10th of the standard cone calorimeter, ISO 5660-1, flow). The concentration of oxygen in the chamber was measured by means of sampling the gas and feeding it into the oxygen-concentration sensor. This sensor was a UV-flux-based sensor type CO2Meter, type CM-42991. Prior to the experiments, the oxygen-concentration sensor was calibrated against a paramagnetic sensor, used in standard cone-calorimeter operations.

At the top of the stack, a specially designed steel mesh holder was mounted to support the exposed CH model samples. On the steel mesh holder, four CH model samples of dimension 13 mm × 76 mm can be simultaneously exposed to fire effluents and high temperatures. Additionally, a gas-sampling probe was installed at the end of the stack to sample the gases produced when burning the spruce blocks. The probe consists of a steel pipe with an inner diameter of 8 mm, sealed at the end and bored with 7 holes of diameter 2 mm, oriented away from the flow of the combustion gases to prevent clogging of the probe.

During the experiment, one set of CH model samples was exposed at the top of the stack, placed on the test-specimen support, as described above. Additional CH model samples were installed in the so-called CH test chamber, which was part of the gas-sampling line, as described below. The CH test chamber is essentially a glass tube, 30 mm in diameter and 200 mm log. The purpose of the CH chamber is to accommodate the test sample in a gas flow during the experiment. During the experiment, the incident heat-flux density was set to 50 ± 0.5 kW/m^2^ for all the experiments. Prior to an experiment the conical heater, providing a constant heat flux, was stabilized and the heat-flux density was calibrated. There was no need to measure the temperature of the burning wood at the surface as the thermal conditions are sufficiently defined by the known incident heat-flux density.

The oxygen concentration, sampled in the same plane and about 3 cm away from the burning wood, was measured, set prior to the experiment and controlled during the experiment to be 15% ± 0.5%. The selected heat-flux density and the selected oxygen concentration represent a fully developed, ventilation-controlled fire.

During the experiment the generated gases were sampled with the sampling probe at the top of the stack. The probe was connected to the pump, providing a constant extraction of the sampled gases. Immediately after the sampling, the tube connected to the sampling probe was split with a t-piece, forming two branches. The first branch—the test-sample branch—incorporated three elements: a sample-holding chamber in which the test sample is placed, a glass-fibre, a 1-µm filter disc for collecting the particles in the sampled flow and a polyurethane adsorbing foam (PUF), Sigma Aldrich, ORBO 1000, to collect the gaseous components of the sampled flow. The second branch—the blank branch—incorporated the same elements, but in a different order: first was the glass-fibre filter, followed by the PUF foam and last was the sample-holding chamber. The chamber was empty, but it was in place to keep the same gas-flow resistance in both branches as close as possible to justify the assumption that half of the total sampled flow was led across the test sample through the test-sample branch.

Additionally, during the experiment the temperature was measured at two places: (1) on the top of the CH stack, just below the exposed sample and (2) inside the CH test chamber. The temperature was measured using a type-k thermocouple connected to a data-acquisition system. The measured temperatures from the experiments involving exposures of the egg yolk and lead white tempera are gathered in Tables 2 and 3, respectively.

**Table 2:** Measured temperatures (°C) on the CH stack and in the CH test chamber during the exposure of the egg yolk (E) samples to fire-related effects in relation to time.

Time (min)	Batch 1 (E)		Batch 2 (E)		Batch 3 (E)	
	Temperature (°C)					
	CH stack	CH test chamber	CH stack	CH test chamber	CH stack	CH test chamber
Empty firebox	199	32	236.9	32.8	238.5	32
Soot mass pump on	180	32.2	207.2	32.7	192.4	32
0 (ignition)	236	33.7	224.4	32.5	318.2	32
0.5	248	35.2	376.6	32.6	331.7	32.6
1	283	36.4	342.9	32.9	319.3	33.,1
3	172	39.6	281.1	34.4	298.2	35.6
5	176	40.9	281.4	35.6	301.6	37.8
7	179	41.2	274.4	36.6	279.2	38.9
10	182	41.7	306.9	37.8	305	39.1
12	185	41.8	350	38.4	345.7	37.5
15	193	42.3	401	37.1	444	36
18	196	42.1	315	36.5	300	35.2
20	188	41.9	271.4	35.9	267.8	34.7

**Table 3:** Measured temperatures (°C) on the CH stack and in the CH test chamber during the exposure of the lead white tempera (E + LW) samples to fire-related effects in relation to time.

Time (min)	Batch 1 (E + LW)		Batch 2 (E + LW)		Batch 3 (E + LW)	
	Temperature (°C)					
	CH stack	CH test chamber	CH stack	CH test chamber	CH stack	CH test chamber
Empty firebox	168	28.5	243.2	31.1	254.2	31.2
Soot mass pump on	182	30.6	176.5	30.9	209.1	31.1
0 (ignition)	283	31.5	367.5	31	382.9	31.2
0.5	355	36.3	351.2	31.4	361.0	31.5
1	332	39.4	322.4	31.9	337.3	31.9
3	302	49.0	283.9	35.1	306.8	33.6
5	256	49.5	270.3	37.8	293.7	35.2
7	254	46.6	281.4	39.6	296	36.6
10	291	43.3	291	41	302.1	38
12	316	40.7	310	42.3	311	38.3
15	406	39.0	365.7	39.6	385.8	38.1
18	225	39.5	360.2	36.8	318.6	38
20	205	38.8	256.1	35.8	248.5	37.8

#### FTIR spectroscopy

##### Transmission mode

Transmission FTIR spectra were recorded with a Perkin Elmer (USA) Spectrum 100 FTIR spectrophotometer coupled to a Spotlight FTIR microscope equipped with a nitrogen-cooled mercury-cadmium-telluride (MCT) detector. The samples taken from the model samples were placed between the windows of a diamond anvil cell and examined under a microscope with an aperture of 100 µm × 100 μm. The spectra were scanned from 4000 to 600 cm^‒1^, using 64 spectral scans per experiment and with a 4 cm^‒1^ spectral resolution. On average, three different transmission FTIR spectra were collected for each sample. All the transmission FTIR spectra were then baseline corrected and averaged using the OPUS 7.0 data-collection software package (Bruker, Germany). We refer the reader to our previous manuscript for the methodology^21^.

##### Reflection mode

Total-reflection Fourier-transform infrared (FTIR) spectra of the cultural heritage model samples were recorded with a portable Bruker Optics (Germany) ALPHA–R spectrometer. The instrumentation is equipped with a dedicated reflection module, which allows contactless and non-destructive FTIR analysis with an uncooled, deuterated, L-alanine-doped, triglycine sulphate (DLaTGS) detector and a 25°/25° optical layout. The integrated video camera provided a view of the sampling area of about 28 mm^2^. Pseudo-absorption spectra (A′ = log(1/R), R = reflectance) of the exposed E + LW model samples were collected in the range 5000–375 cm^‒1^, while in the case of the E model samples, the pseudo-absorption spectra were collected in a narrower range from 5000 to 1250 cm^‒1^. Namely, in the corresponding total-reflection spectra, the wavenumber range below 1250 cm^‒1^ is affected by the characteristic bands of the glass slide on which the model samples were applied. The spectral resolution was 4 cm^–1^ and 160 scans were collected for one spectrum. The background was acquired on a gold mirror.

On average, three different reflection spectra were collected for each sample. All the reflection FTIR spectra were then averaged using the OPUS 7.0 data-collection software package (Bruker, Germany). We refer the reader to our previous manuscript for the methodology^22^.

#### Raman spectroscopy

Samples on the glass slides were placed under the microscope and analysed or a small part was scratched off and then analysed. The Raman spectra were recorded with a Horiba Jobin Yvon LabRAM HR800 Raman spectrometer coupled to an Olympus BXFM optical microscope. The spectra were collected using a 785-nm laser excitation line, a macro lens, a × 100 or × 50 long-working-distance objective lens, and a 600-grooves/mm grating. A multi-channel, air-cooled CCD detector was used. The experimental conditions were adjusted according to the characteristics of the samples.

#### Colourimetry

Changes in the colour of the CH samples were investigated with a colourimeter type i1PRO (X-Rite Inc., USA), using the GretagMacbeth colour-management system. A D65 light source, an observation angle of 10° and 4.5-mm aperture were used. Each microscope slide with dried sample material was measured for its colour before and after the exposure to fire-related effects (every time on the same three different parts of the sample). Computations were performed in the CIE L*a*b* colour space^23^.

#### Pyrolysis GC MS

The incorporation of a pyrolysis unit into the combined gas-chromatography mass-spectrometry system enabled a study of the non-soluble, non-volatile, often solid-state samples, as it can turn them into volatile products by heating them up to very high temperatures (up to 1400 °C) in a very short time (8 ms), in an inert atmosphere, causing degradation of the sample into volatile products. The pyrolysis reaction products are characteristic of the original structure of the sample^24^. Rapid heating was used to decrease the risk of secondary reactions.

A powdered sample was prepared by removing the paint layer from the glass microscope slides with a scalpel. The powder was divided into two samples. One sample was measured without further treatments by pyrolysis GC–MS. The other sample was derivatised with BSTFA:TMCS (99:1) before the measurements to make possible the detection of more polar compounds.

The pyrolysis was performed using a foil pulse pyrolyser (Pyrola 2000, Pyrol AB, Lund, Sweden) with a chamber temperature of 150 °C, a pyrolysis temperature of 550 °C, a temperature rise time of 8 ms and a total pyrolysis time of 2 s. The pyrolyser was connected to an Agilent 5890 gas chromatograph (split injector: split ratio 1:10 and temperature 250 °C). The capillary GC analysis was performed on an HP5-MS (30 m × 0.25 mm id, 0.25 µm) capillary column with helium as the carrier gas. The GC starting temperature was 50 °C, held for 1.5 min, then gradually increased to 300 °C (rate of 10 °C min^−1^, carrier gas flow 1.2 ml min^−1^). The eluting compounds were detected with an Agilent 5977B High Efficiency Source (HES) single-quadrupole mass detector. The transfer line, ion source, and quadrupole analyser temperatures were maintained at 290 °C, 230 °C, and 150 °C, respectively. In the full-scan mode, the electron impact ionization (EI) mass spectra in the range 33–600 (m/z) were recorded at an electron energy of 70 eV.

## Results and discussion

The following sections present the changes in the molecular structures for the exposed samples of egg yolk adhesive (E) and lead white tempera (E + LW) due to fire-related effects using non-invasive reflection and transmission FTIR spectroscopy, Raman spectroscopy and pyrolysis GC–MS. In addition, in each section, colour changes to the CH samples exposed to fire-related effects are evaluated using colourimetry. The results for the egg yolk model samples collected by means of conventional and non-invasive FTIR spectroscopy are very similar for batch 2 and batch 3. Therefore, in the section “Egg yolk adhesive” the molecular changes of the proteinaceous binder (E) from batch 1 and batch 2 are described, whereas the results obtained from batch 3 are not included. In the case of the lead white tempera (E + LW), the molecular changes identified in the infrared spectral analyses were the same or comparable for all three batches. Therefore, only the results from batch 1 are presented in the Section “Lead white tempera”.

### Egg yolk adhesive

#### Transmission FTIR spectroscopy

**Figure 2:**
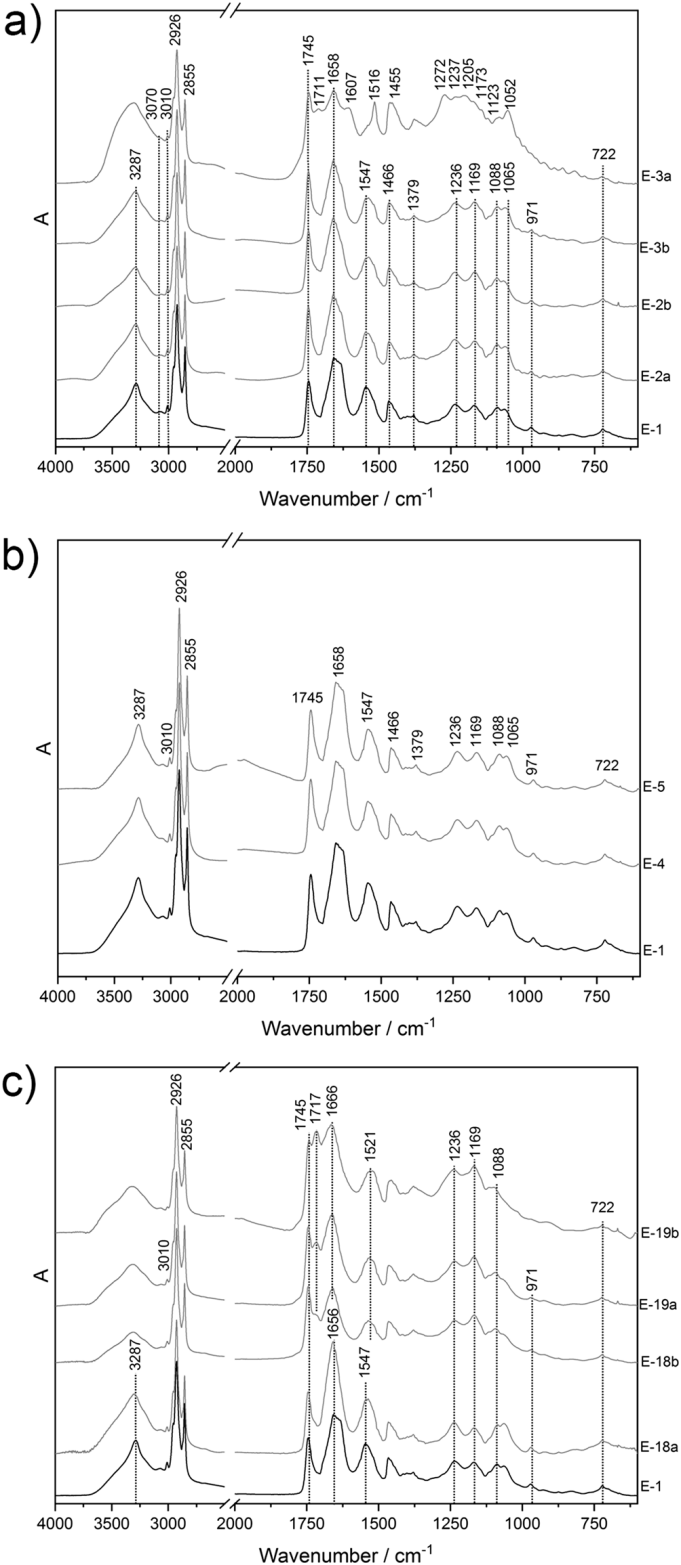
Transmission FTIR spectra of exposed egg yolk (E) model samples: (**a**) (batch 1) on CH stack for different times (20 min—3a; 5 min—3b; 1 min—2b; 30 s—2a; 0 s—1); (**b**) (batch 1) in CH test chamber ( 20 min—4, 5; 0 s—1) and (**c**) (batch 2) on CH stack for different times (20 min—19b; 5 min—19a; 1 min—18b; 30 s—18a; 0 s—1).

Transmission FTIR spectra of the egg yolk (E) model samples from batch 1, exposed to fire-related effects on top of the CH stack, are presented in Fig. 2a. The spectrum of the reference proteinaceous model sample (E-1) exhibits all the characteristic bands of egg yolk. All these distinctive bands of egg yolk adhesive are also visible in the spectra of the model samples E, exposed for 30 s (E-2a), 1 min (E-2b) and 5 min (E-3b). Interestingly, no molecular changes were observed with the transmission FTIR spectroscopy. On the other hand, the effects of the fire effluents and the increased temperature are visible in the spectrum of the egg yolk sample (E-3a) that was exposed for the longest time (20 min). The newly formed sharp signal at 1516 cm^−1^ belongs to an in-plane mode of the aromatic ring of a tyrosine residue^25,26^. Tyrosine is one of the four aromatic amino acids, and due to its polar character it is a very strong infrared absorber^27^. In the E-3a spectrum the signals at 1607 (ν(CC)ring, δ(CH)), 1455 (δ(CH_2_)), 1272 (ν(CO), ν(CC)), 1237 cm^‒^^1^ (δ(COH)) are the characteristic bands of tyrosine amino acid^27^. Moreover, in this transmission spectrum some additional peaks could belong to different aromatic compounds (e.g., p-cresol, phenol, and 4-methylphenol) and are at 1610, 1272, 1205, 1173, 1123 and 1052 cm^‒1^^28,29^. Most likely, the signals in the lower-medium IR region apparently belong to vibrations of the arene ring, substituted with different molecular groups. Additionally, the newly formed spectral feature at 1711 cm^‒1^ belongs to the free fatty acids.

Figure 2b illustrates the transmission FTIR spectra collected from egg yolk adhesive exposed to fire effluents in the CH test chamber (batch 1). The mentioned spectra exhibit distinctive signals of non-denatured proteinaceous binder (3287, 3010, 2926, 2855, 1745, 1658, 1547, 1466, 1379, 1236, 1169, 1088, 1065, 971 and 722 cm^‒1^), meaning that neither the temperature nor the emission of fire effluents induced molecular changes detectable by vibrational spectroscopy. As seen in Table 2, the highest temperature in the CH test chambers (batch 1, batch 2 and batch 3) did not exceeded 42 °C. This temperature is not high enough for egg yolk proteins to be subjected to a thermal shock. Indeed, egg yolk proteins are not subjected to denaturation processes when heated to less than 69 °C^30^.

The transmission FTIR spectra of the proteinaceous model samples from batch 2, exposed to fire-related effects on the CH stack, are compared in Fig. 2c. In the FTIR spectrum of the model sample with the shortest time of exposure (i.e., 30 s) to fire-related effects, no spectral differences were observed in comparison to the spectrum of the pure egg yolk sample (E-1). The first molecular changes were observed in the spectra after one minute of exposure (E-18b). In this transmission FTIR spectrum the oxidation of the egg yolk triglyceride units is indicated as broadening of the carbonyl stretching vibration at 1745 cm^‒1^ along with the formation of a new shoulder band at 1717 cm^‒1^. Molecular changes caused by the oxidation of triglycerides are distinctly more visible in FTIR spectra acquired from the proteinaceous model samples exposed to fire-related effects for longer times (E-19a, E-19b). Namely, the intensity of the absorption band of the ν (C=O) vibration of carboxylic acids at 1717 cm^‒1^ drastically increased and in the spectrum of the E-19b model sample, the intensity of this band becomes even higher than the intensity of the characteristic lipid band at 1745 cm^‒1^. The broadening and loss of intensity of the bands in relation to the time of exposure to fire-related effects are more visible in the C-O stretching pattern at 1236, 1169 and 1088 cm^‒1^. Furthermore, the Amide I and Amide II bands in the transmission FTIR spectra collected on the egg yolk model samples exposed to fire-related effects for 1, 5 and 20 min are shifted by ~ 10 cm^‒1^ to higher and ~ 25 cm^‒1^ to lower wavenumbers, respectively. The mentioned shifting of the Amide bands indicates changes to the protein’s secondary structures (i.e., α-helices and β-sheets)^31^. Additional spectral modifications are also visible in the NH stretching region, such as broadening of the characteristic Amide A band at 3287 cm^‒1^.

Differences in the molecular changes of the egg yolk adhesive between the batches are probably present due to the exposure to different absolute temperatures in specific batches. Namely, the highest temperature in batch 1 did not exceed ~ 283 °C, whereas the highest temperatures in batches 2 and 3 were ~ 401 °C and ~ 444 °C, respectively.

#### Total reflection FTIR spectroscopy

**Figure 3:**
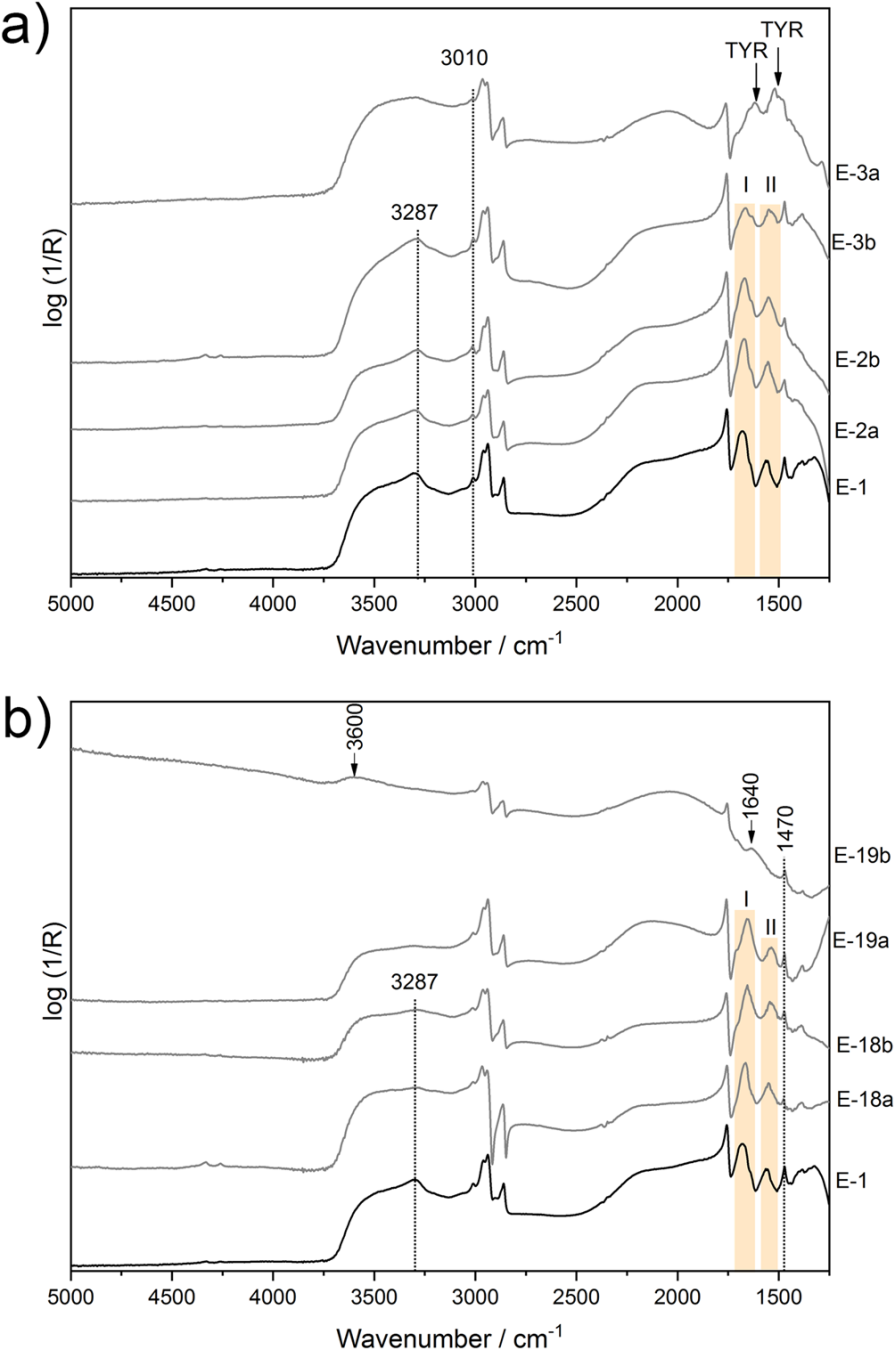
Reflection FTIR spectra of exposed egg yolk model samples (E) to fire-related effects on the CH stack for different times (20 min—3a and 19b; 5 min—3b and 19a; 1 min—2b and 18b; 30 s—2a and 18a; 0 s—1) from (**a**) batch1 and (**b**) batch 2. Labels: TYR-tyrosine, I–Amide I, II- Amide II.

The comparison of the reflection FTIR spectra of the proteinaceous model samples (E) exposed to fire-related effects on CH stack at different times from batch 1 and batch 2 are reported in Fig. 3a and Fig. 3b, respectively. The results acquired from the E model samples in the CH test chamber (batch 1, batch 2 and batch 3) are consistent with the results gathered from the reference spectrum of the egg yolk and are therefore not presented.

Chemical changes on a molecular level are observed according to the main protein bands in the spectra collected from batch 1. In more in details, the Amide I and Amide II bands decreased in intensity in relation to the time of exposure to the FE. Changes to the band shape are also visible in the region of the Amide A bands. This signal is broadened and in the spectrum of E-3a the maximum at 3287 cm^‒1^ completely vanished. Moreover, a loss of intensity is also observed in the signal of the olefinic C-H stretching of the unsaturated fatty esters of the egg yolk at 3010 cm^‒1^. Furthermore, the reflection FTIR spectrum of the E model sample from batch 1 exposed for the longest time (E-3a) shows distorted spectral features at 1620–1590 and 1520–1505 cm^‒1^. These are the characteristic bands of tyrosine amino acid (see Fig. 3a, spectrum E-3a). In comparison with the transmission FTIR (Fig. 2), the identification of the tyrosine amino acid by means of non-invasive reflection FTIR spectroscopy is not so straightforward. Especially because the sample was covered by soot, which impacts on the penetration of the IR beam.

The characterisation of the molecular changes of the E model samples from batch 2 was, in the case of the sample E-19b that was exposed for the longest time to fire-related effects, hampered by the formation of a new, additional layer of a black-powder deposit resulting from the wood burning. This extra layer, which is also present on the other exposed model samples (batch 3), contains small particles that include soot. In the total-reflection spectrum of E-19b, the characteristic protein bands (i.e., Amide I, Amide II, Amide A) are no longer visible (Fig. 3b). The strong carbonyl stretching vibration at 1760–1720 cm^‒1^ is still evident in the E-19b spectrum, whereas the band at 1470 cm^‒1^ remains unchanged in all the total-reflection spectra of the proteinaceous samples exposed to fire-related effects (E-18a, E-18b, E-19a, and E-19b) acquired from batch 2. The broadened signal with a maximum at 1640 cm^‒1^ could be the residue of the Amide I band or even more likely this band could belong to the C=C vibrations of the aromatic moieties resulting from the burning of organic matter^22^. Furthermore, significant shifting is observed in the OH stretching region. The broad band with a maximum at ~ 3600 cm^‒1^ suggests the possibility of OH–OH vibrations of adsorbed water from the uppermost soot layer^32^.

In the reflection FTIR spectra of the samples exposed to fire-related effects on the CH stack for the shortest time (E-18a, E-18b, E-19a), the Amide I band has slight broadening related to the time of the samples’ exposure, whereas the Amide II band is losing its relative intensity. As was already mentioned for batch 1, the band of Amide A at 3287 cm^‒1^ is broadening in the spectra acquired from the CH samples exposed for 30 s and 1 min and in the spectrum of E-19a it has completely vanished.

#### Pyrolysis GC–MS

Pyrolysis of the egg yolk (E-1) and the egg yolk model samples from batches 1 and 2 (E-3a and E-19b), which were exposed for the longest time to the effects of fire effluents and increased temperatures on top of the CH stack, were performed, and followed by detection in the GC–MS. A list of the pyrolysis reaction products of these samples can be found in Supplementary Tables S1 (detected pyrolysis degradation products) and S2 (extra detected pyrolysis degradation products after derivatization of the sample). The pyrolysis-GC–MS (py-GC–MS) analysis technique was used to confirm and complement the results that were obtained using vibrational spectroscopies.

##### Py-GC–MS of egg yolk reference (E-1)

The major compounds obtained from the fast pyrolysis of the lipids were carboxylic acids, esters, aliphatic hydrocarbons, 1-alkenes and CO_2_ (see Supplementary Table S1 and S2). Conversely, pyrolyzed proteins are challenging to detect, as the amino acids (their building blocks) yield rapidly-eluting, volatile fragments, which are difficult to analyse with gas chromatography^33^. Therefore, it was especially important to find specific markers that enable the detection of certain amino acids^24,33,34^. Aromatic amino acids can be reliably detected with pyrolysis-GC–MS, as their pyrolysis degradation products are well-separated by the chromatography column and captured by the mass detector, and therefore it is possible to accurately reconstitute the information about the original amino acids’ presence. The indole type of compounds are characteristic decomposition products of tryptophan; benzyl cyanide, toluene and styrene can be used to detect phenylalanine, and the sum of the phenol and substituted phenols can be used for the detection of tyrosine^24,34,35^. Therefore, in Supplementary Tables S1 and S2, it is shown that the phenol, cresol, dimethyl phenol, methoxy phenol and ethyl phenol reaction products are obtained from the pyrolysis of tyrosine; indole and methyl-indole are characteristic for tryptophan; while toluene, styrene and benzyl nitrile come from phenylalanine. Other N-containing compounds, like acetamide and pyrrole, also resulted from the decomposition of proteins. The interaction of the long C-chain compounds of lipids (e.g., hexadecanoic acid) with N-containing compounds of proteins during the pyrolysis resulted in the formation of long-chain nitriles from the interaction. These results are in accordance with the findings of Gautam and Vinu^36^. The pyrolysis of carbohydrates was characterized by the formation of furanose.

##### Py-GC–MS of CH stack-exposed egg yolk adhesive (E-3a and E-19b)

The first observed difference between the reference model sample E-1 and the egg yolk adhesive, exposed on the CH stack (E-3a and E-19b), was that the concentration of detected pyrolysis reaction products, which are characteristic for lipids (e.g., carboxylic acids, aliphatic hydrocarbons, 1-alkenes and CO_2_), was much lower in the exposed egg yolk adhesive (E-3a and E-19b) than in the reference (E-1). This can be easily explained by the lipids’ propensity to polymerize under the influence of heat^37^ via thermolytic reactions^38^. This has a negative effect on the degradation of lipids into their pyrolysis reaction products, which can impede the detection in the GC–MS. The reason is that, in general, low-molecular-weight molecules have a much higher chance to volatilize (and therefore be able to enter the GC–MS analysis) by pyrolysis, instead of remaining in the solid matrix and further polymerize to high-molecular-weight products, eventually turning to char^39^.

The differences in the proteins’ composition between the reference model sample E-1 and the CH stack-exposed egg yolk adhesive (E-3a and E-19b) are discussed, with a focus on the aromatic amino acids (tyrosine, tryptophan and phenylalanine). It is widely known that heat causes the denaturation of proteins. Furthermore, tryptophan and tyrosine buried within the core of the protein molecule in its native state might be exposed during the denaturation^40^. In our study these effects were detected using FTIR spectroscopy (appearance of an additional band at 1516 cm^‒1^, characteristic for tyrosine) (see section “Transmission FTIR”) and also confirmed with the py-GC–MS. As described above, a large range of pyrolysis reaction products of tyrosine was obtained for the reference sample E-1. This is due to interactions with the neighbouring amino acids, while the tyrosine residues were still compacted in the core of the protein. However, in the samples of exposed egg yolk adhesive, only cresol and phenol were found as the pyrolysis reaction products, signalling a reduction in the interactions with other amino acids (tyrosine “freed”). On the other hand, during heat exposure, crosslinking of the denatured protein chains can also happen, which can be observed in the results presented in Supplementary Tables S1 and S2, when comparing the reference sample E-1 with the burned egg yolk E-3a and E-19b. The strong interactions that occur during the crosslinking cause that protein pyrolysates, which now contain stronger bonds, are kept in the char that is formed in abundance in the protein pyrolysis. In this way, much-less-volatile pyrolysis reaction products are formed in the exposed egg yolk adhesive samples E-3a and E-19b^35^.

Egg yolk also contains a small percentage of carbohydrates. It is widely known that heating causes the isomerisation and decomposition of carbohydrates via the caramelization or Maillard reaction. Isomerization of the carbohydrates in the exposed egg yolk adhesive samples (E-3a and E-19b), in comparison with the model reference sample E-1, can be observed with pyrolysis due to a change in the pyrolysis reaction products. In the reference sample E-1, the main product is a furanose. The CH stack-exposed egg yolk adhesive samples (E-3a and E-19b) have pyranose and levoglucosan pyrolysis reaction products. The decomposition of the carbohydrates is also seen through lower concentrations of the carbohydrate pyrolysis reaction products in the exposed egg yolk samples (E-3a and E-19b).

The differences observed between the E-3a and E-19b model samples are most probably due to the higher absolute temperature reached in batch 2 and 3 in comparison with batch 1, as well as the longer exposure time to these higher temperature in batches 2 and 3 (see Table 2). Since E-19b was exposed to high temperatures for a longer time, it seems that the effects of the polymerization and crosslinking reactions in lipids and proteins were already more pronounced, leading to a decrease in their pyrolysis reaction products.

#### Colourimetry

**Figure 4:**
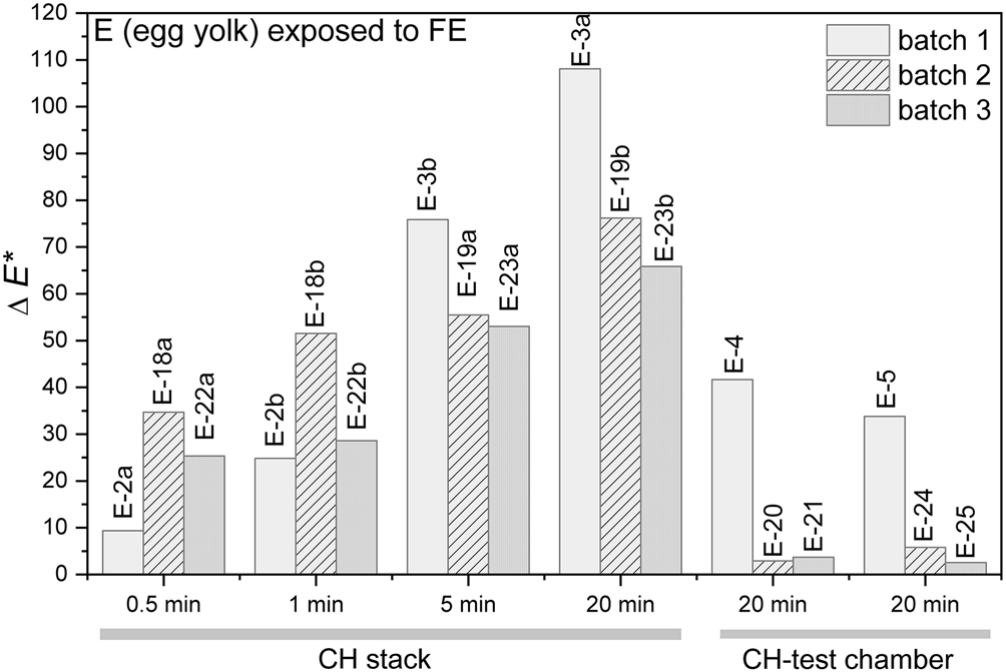
Total colour change (ΔE*) for the egg yolk (E) samples exposed to fire-related effects (FE) on the CH stack for different times and in the CH test chamber for all batches.

The delta values of the parameters (∆L^*^, ∆a^*^ and ∆b^*^) and the total colour changes (ΔE^*^) for the samples of egg yolk (E) from batches 1, 2, 3 in both experiments, on the CH stack and in CH test chamber, were calculated and are presented in Table 4 and Fig. 4. The changes in the colour were more pronounced on the samples placed on the CH stack and were already visible to the naked eye on the samples exposed for only 30 s. The value of the total colour change increased in relation to the longer exposure times (Fig. 4). Namely, the value of 9.4/34.7/25.3 calculated for the samples exposed for 30 s (E-2a, 18a, 22a) increased to 108.1/76.2/65.9 for the samples exposed for 20 min (E-3a, 19b,23b). The largest differences contributing to the total colour difference/change were calculated for the ∆L^*^ and *∆b, *meaning that the primary effects were darkening and yellowing of the samples. The colour change was much less noticeable for the samples placed in the CH test chamber, especially in the case of batch 2 and batch 3 (samples E-20, 21, 25). In general, the values of batch 1 deviate from the values of the batches 2 and 3. On the other hand, the values calculated for batch 2 and 3 are more comparable.

**Table 4:** Delta values (∆L^*^, ∆a^*^ and ∆b^*^) and calculated total colour change (ΔE^*^) for the samples of egg yolk adhesive (E) exposed to fire-related effects for different times on the CH stack and in the CH test chamber.

Sample	∆L*	∆a*	∆b*	ΔE*	Sample	∆L*	∆a*	Δb*	∆E*	Sample	∆L*	∆a*	Δb*	∆E*
E-2a	~ 0.0	− 1.3	9.3 9.27	9.4	E-18a	− 30.8	~ 0.0	16.0	34.7	E-22a	− 21.1	0.2	13.9	25.3
E-2b	− 15.5	0.4	19.4	24.8	E-18b	− 42.5	2.7	29.0	51.5	E-22b	− 23.6	0.6	16.1	28.6
E-3b	− 54.8	2.9	52.4	75.9	E-19a	− 47.3	4.4	28.7	55.5	E-23a	− 41.8	4.5	32.4	53.0
E-3a	− 74.5	16.8	76.4	108.1	E-19b	− 75.8	− 1.2	7.9	76.2	E-23b	− 65.3	− 1.6	8.3	65.9
E-4	− 31.3	− 2.3	27.4	41.7	E-20	0.8	− 0.7	2.7	2.9	E-24	− 0.1	− 0.8	3.6	3.7
E-5	− 29.2	− 1.2	17.0	33.8	E-21	− 0.7	− 1.0	5.7	5.8	E-25	0.6	− 0.7	2.4	2.5

### Lead white tempera

#### Transmission FTIR spectroscopy

**Figure 5:**
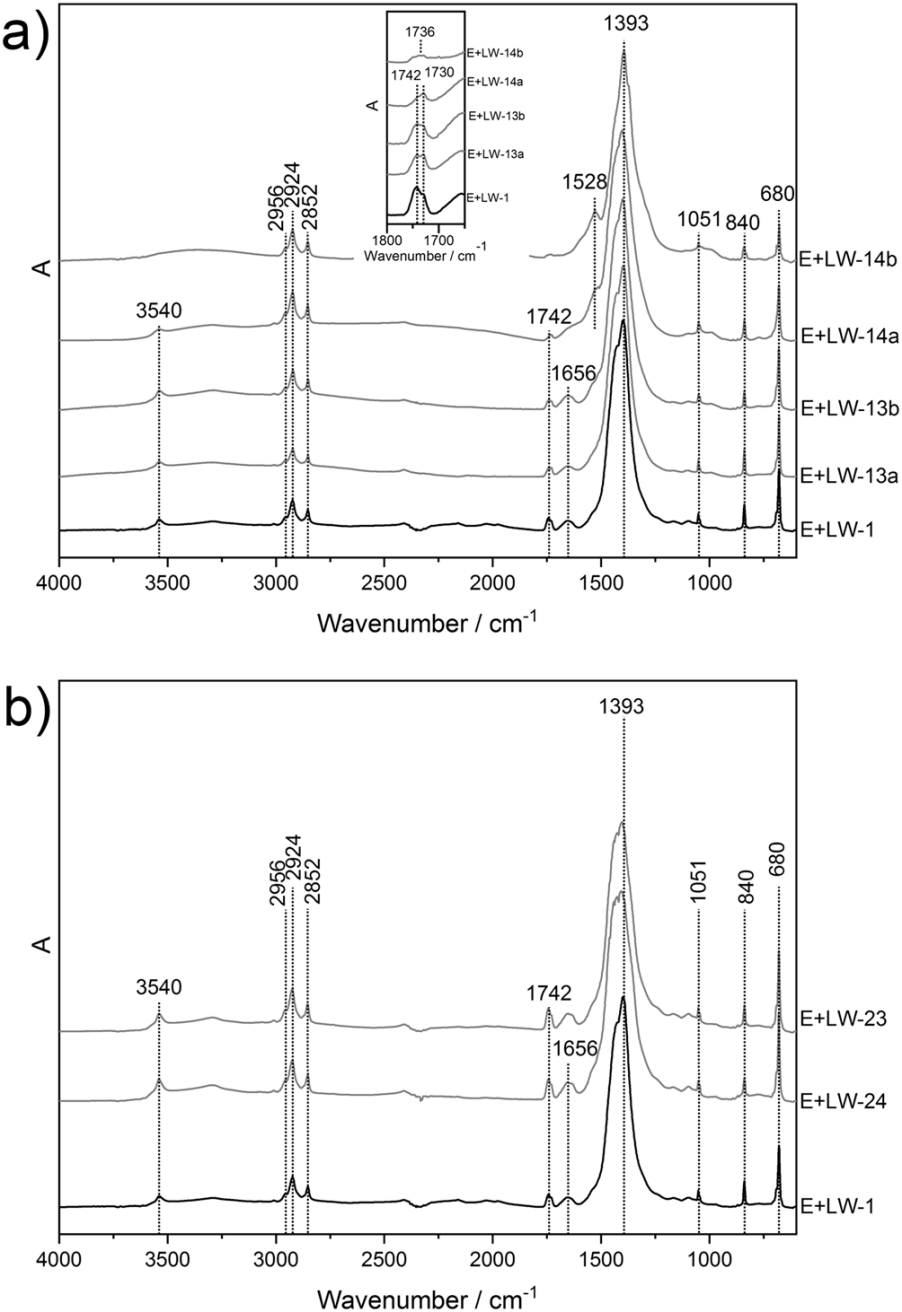
Transmission FTIR spectra of exposed CH samples (BATCH 1) for different times (20 min—14b; 5 min—14a; 1 min—13b; 30 s—13a; 0 s—1 (**a**) on CH stack and (**b**) CH-test chamber. The inset indicates a region of interest.

The transmission FTIR spectra of the lead white tempera (E + LW) exposed to fire effluents and increased temperatures on the CH stack for different times of exposure and the unexposed E + LW sample are presented in Fig. 5a. The IR spectra collected from the E + LW samples exposed to fire-related effects for 30 s and 1 min are in good agreement with the transmission spectrum of the unexposed sample E + LW. In all the aforementioned IR spectra, no changes in the positions of the characteristic bands of pigment lead white and egg yolk binder as well as no differences in their intensities were detected. As expected for the pigment–binder mixture, the strongest signal of the CO_3_ stretching vibration of the pigment lead white at 1393 cm^‒1^ hindered the typical egg yolk band of Amide II at ~ 1550 cm^‒1^^41^.

Furthermore, noticeable changes on a molecular level were observed in the transmission FTIR spectra recorded on the E + LW samples exposed to fire-related effects for longer times (i.e., 5 min and 20 min) in Fig. 5a. The intensity of the significant lead white band of the hydroxyl stretching vibration at 3540 cm^‒1^ is reduced and in the IR spectra of the sample exposed for 20 min it completely disappears. The same observations can be made in the region between 1745 and 1730 cm^‒1^. The carbonyl stretching vibration of the egg yolk (1742 cm^‒1^) decreases in intensity in the spectra collected on samples with longer exposures. The adjacent combination band (ν_1_ + ν_4_) of the pigment lead white at 1730 cm^‒1^ maintains its intensity in the IR spectra collected from the E + LW samples with up to 5 min of exposure. In the FTIR spectrum of the sample after 20 min of exposure, the doublet of the mentioned binder and pigment vibrations is no longer visible, but a very weak and broadened signal is still present with maximum at 1736 cm^‒1^ (inset Fig. 5a). In addition, the characteristic Amide I band of egg yolk is no longer visible in the spectra collected on the samples with a longer exposure to fire-related effects (E + LW-14a, E + LW-14b). In those spectra, the strongest signal of the carbonate anion stretching vibration is broadened and an additional band at 1528 cm^‒1^ appears. This newly formed signal, which is clearly seen in the spectrum of the E + LW sample exposed to fire-related effects for 20 min, most probably belongs to the asymmetric stretching vibrations of the COO-group of lead soaps. It is known, that the deterioration of artworks affected by different impacts (i.e., environmental conditions, fire effluents, etc.) could cause the formation of free fatty acids in lipidic and in a proteinaceous medium as well^42^. A heavy-metal cation from the pigment (i.e., lead) reacts with the mentioned free fatty acids and the results are metal soaps, which can then form few-hundred-micrometres-large aggregates or inclusions^43^.

The transmission spectra collected from the exposed E + LW samples in the CH test chamber presented in Fig. 5b have similar spectral characteristics to the IR spectra of the unexposed E + LW sample. It is worth mentioning that the highest temperature in the CH test chamber was below 50 °C throughout the experiment (see Table 3). This observation can lead to the conclusion that high temperatures have a significantly greater influence on the molecular structure of the lead white tempera samples on the CH stack than the influence of the fire effluents themselves.

#### Total reflection FTIR spectroscopy

**Figure 6:**
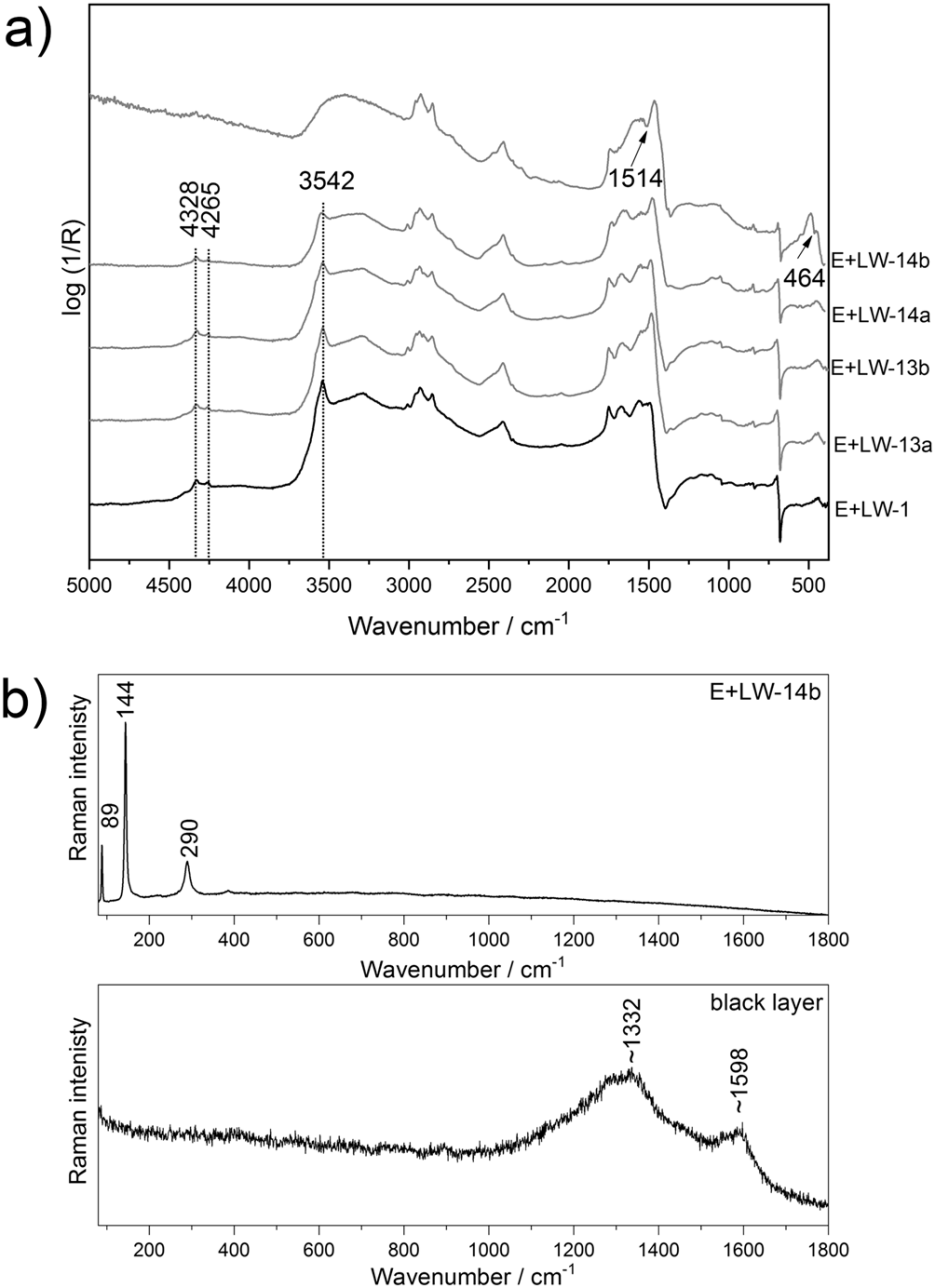
(**a**) Reflection FTIR spectra of lead white tempera model samples (E + LW) exposed to fire-related effects (BATCH 1) for different times (20 min—14b; 5 min—14a; 1 min—13b; 30 s—13a; 0 s—1) on CH stack. (**b**) Raman spectra collected from the sample E + LW-14b, identifying PbO (upper spectrum) and from the black layer (soot), identifying carbon bands (lower spectrum).

The total reflection FTIR spectra of the E + LW samples exposed to fire-related effects are presented in Fig. 6a. The reflection FTIR spectra collected on the CH samples (E + LW-23 and E + LW-24) from the CH test chamber are comparable to the spectrum of the reference E + LW-1 sample and are not included in the manuscript. Furthermore, the results of the total reflection FTIR technique are in good agreement with the results from conventional transmission FTIR spectroscopy. The analysis of the E + LW samples after longer exposures to fire-related effects on the CH stack revealed a partial pigment dissociation. Namely, in the reflection spectrum collected on the E + LW sample exposed for 20 min (E + LW-14b) to fire-related effects, a distinctive band of lead monoxide at 464 cm^−1^^44^ is visible in addition to the lead carbonate signals. Lead monoxide is a well-known thermal degradation product of different lead carbonates. The presence of PbO was confirmed using Raman spectroscopy (see Fig. 6b) based on its characteristic Raman bands at 89, 144, 290 cm^‒1^^45^. Furthermore, on the sample E + LW-14b, a dark deposition layer (soot) on the edge of the sample on the glass side was analysed using Raman spectroscopy (Fig. 6b), where bands of carbon (broad bands (D, G) at ~ 1332, 1598 cm^‒1^) were detected^46^. Many Raman measurements were affected by a strong fluorescence interfering with the Raman signal under the selected conditions. It seems that the interfering fluorescence effect increased in the spectra of the samples with longer exposures (Supplementary Fig. S1), since the lead white was also more difficult to detect based on its characteristic band at ~ 1056 cm^‒1^^45^. Moreover, in the total reflection spectrum of E + LW-14b, the distinctive signal of lead carboxylate at 1514 cm^‒1^ was also observed. The dehydration of the E + LW-14b sample is confirmed by the disappearance of the sharp band of the hydroxyl stretching vibration at 3542 cm^‒1^. The characteristic bands of the proteinaceous binder decreased in intensity in the spectra collected from the E + LW samples with longer exposures and in the spectrum collected from the 20-min-exposed sample it completely vanished. In the near-IR region a peculiar combination of ν(NH) + δ(NH) and ν(OH) + δ(OH) bands of the lipidic component of the egg yolk binder at 4328 and 4265 cm^‒1^ are no longer visible in the spectrum of E + LW-14b, suggesting the decomposition of the lipidic compounds.

#### Pyrolysis GC–MS

The pyrolysis of lead white tempera reference (E + LW-1) and lead white tempera model samples from batch 1 and 2 (E + LW-14b and E + LW-32b), which were exposed for the longest to the effects of fire effluents and the increased temperature on top of the CH stack, was performed. A list of the pyrolysis reaction products of these samples can be found in Supplementary Tables S3 (detected pyrolysis degradation products) and S4 (extra detected pyrolysis-degradation products after the derivatization of the sample) (supplement).

##### *Py-GC–MS of lead white tempera (E* + *LW-1)*

Lead white tempera (E + LW) behaves completely differently during pyrolysis than the pure egg yolk adhesive (E). It appears that even in a very short time of the pyrolysis process, oxidation of the sample occurs due to the presence of the pigment lead white^47^. This can be mainly seen by the formation of aldehydes in the lipid pyrolysis reaction products^48,49^, such as decanal, dodecanal, tridecanal, tetradecanal, pentadecanal, hexadecanal, hexadecenal, heptadecanal, heptadecenal, octadecanal, octadecadienal and octadecenal, which were detected in large quantities (see Supplementary Table S3).

It is also apparent that the mechanism of pyrolysis of the proteins in these samples was the formation of radicals. These reactive chemical species can attack proteins (backbone and amino acid functional groups), leading to the formation of protein carbonyls^50^. The indirect damage of proteins by secondary by-products (oxidative modified sugars, aldehydes and lipids) can also occur^51^. In higher-ordered structures, the peptide backbone seems to be protected, since the side chains provide initial targets for the radicals. The aromatic amino acids phenylalanine, tyrosine, and tryptophan are more susceptible to transformation by radicals than others^51^. In Supplementary Tables S3 and S4 for sample E + LW-1, this can be seen by the formation of more low-weight pyrolysis reaction products tht are characteristic for aromatic amino acids: benzene and toluene for phenylalanine; phenol for tyrosine; and indole for tryptophan. The fragments of the broken proteins react with the fatty acid and form long-chain nitriles (octadecynenitrile, octadecenenitrile, hexadecanenitrile) and amides (hexadecanamide).

The analysis of the py-GC–MS results shows that there are no carbohydrate fragments present (unlike in the results for the egg yolk adhesives (Supplementary Tables S1 and S2)), which indicates that oxidative (due to the presence of the lead white pigment) pyrolysis has turned them into CO_2_ and low-molecular-weight pyrolysis reaction products, which are undetectable by GC–MS (Mw < 33 g/mol).

##### *Py-GC–MS of CH stack-exposed lead white tempera (E* + *LW-14b and E* + *LW-32b)*

Radicals are also an essential chemical species in flames. Although present in low concentrations, they are responsible for the ignition and for the rapid reaction rates observed in flames^52^. It is therefore expected that similar (albeit to a lesser extent) oxidative processes as described for pyrolysis above happen during the exposure of the sample to increased temperatures in the fire effluents, leading to degradation of the sample.

For lipids, the lower-molecular-weight pyrolysis reaction products are present in similar concentrations in the lead white reference (E + LW-1) and exposed lead white tempera (E + LW-14b and E + LW-32b) samples. The higher-molecular-weight products, however, are only present in the lead white reference sample E + LW-1. This could be because during exposure to high heat in the fire effluents a set of oxygenated fatty acids is generated, including epoxy-, keto-, and hydroxyl fatty acids (epoxytetradecenol acetate, nonadecanone, heptadecanone hentriacontanone) (see Supplementary Tables S3 and S4)^48,49^. B-scission at the carbonyl- or the alkyl side of the oxygen-bearing carbon atom in the fatty-acid chain will lead to shorter-chain fatty acids^48^.

Unfortunately, because of the destructive character of the oxidative pyrolysis, it is not possible to conclude from the data whether there are any differences in the protein structure between the lead white tempera reference (E + LW-1) and the exposed lead white tempera (E + LW-14b and E + LW-32b) samples.

#### Colourimetry

**Figure 7:**
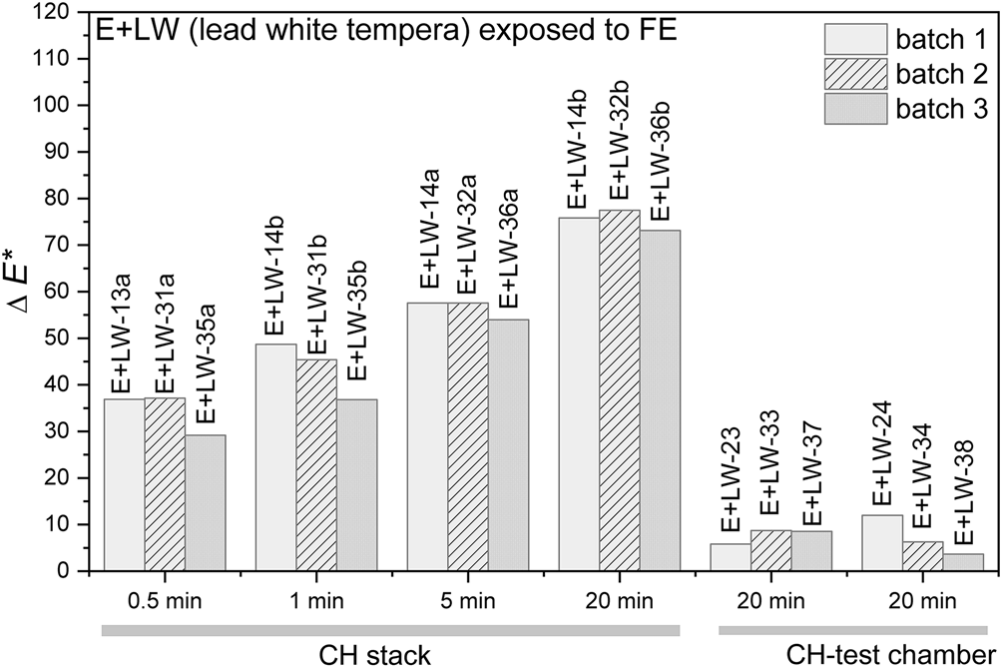
Total colour change (ΔE*) for the lead white tempera (E + LW) samples exposed to fire-related effects (FE) on the CH stack for different times and in the CH test chamber.

The results obtained using colourimetry on the lead white egg tempera (E + LW) exposed to fire-related effects for different times (E + LW-13a–E + LW-14b) on the CH stack and on samples exposed for 20 min in the CH test chamber are presented in Table 5 and Fig. 7. For the samples placed on the CH stack, the total colour change (ΔE^*^) increased in relation to the longer exposure times; it increased from 36.9/37.1/29.1 to 75.8/77.5/73.1 for the samples exposed for the longest time. The largest absolute differences were calculated for the lightness parameter (∆L^*^) and it is related to darkening (negative values of ∆L^*^). For the samples placed in the CH test chamber (E + LW-23, 24, 33, 34, 37, 38), the total colour change is not as high (5.8/12.0/8.7/6.3/8.5/3.7), but in general still high enough to be noticeable to the naked eye^23^. In general and in comparison with the values calculated for egg yolk samples (Table 4 and Fig. 4), the delta values and calculated total colour change are more comparable between the batches.

**Table 5:** Delta values (∆L^*^, ∆a^*^ and ∆b^*^) and calculated total colour change (ΔE^*^) for the samples lead white tempera (E + LW) exposed to fire-related effects for different times on CH stack and in the CH test chamber.

Sample	∆L*	∆a*	∆b*	ΔE*	Sample	∆L*	∆a*	Δb*	∆E*	Sample	∆L*	∆a*	Δb*	∆E*
E + LW-13a	− 35.9	2.9	8.0	36.9	E + LW-31a	− 36.2	2.8	7.8	37.1	E + LW-35a	− 27.2	3.4	10.0	29.2
E + LW-13b	− 48.1	4.5	5.8	48.7	E + LW-31b	− 44.6	4.4	7.2	45.4	E + LW-35b	− 35.4	4.1	9.1	36.8
E + LW-14a	− 57.3	4.5	2.6	57.5	E + LW-32a	− 57.4	4.5	1.6	57.6	E + LW-36a	− 53.3	5.7	6.1	54.0
E + LW-14b	− 75.4	− 0.7	− 7.9	75.8	E + LW-32b	− 76.9	− 1.0	− 9.2	77.5	E + LW-36b	− 72.9	0.3	− 5.3	73.1
E + LW-23	− 2.9	− 0.3	5.0	5.8	E + LW-33	− 2.2	0.4	8.4	8.7	E + LW-37	− 6.8	− 0.2	5.2	8.5
E + LW-24	− 9.1	0.7	7.7	12.0	E + LW-34	− 1.3	− 0.2	6.1	6.3	E + LW-38	0.1	− 1.0	3.5	3.7

## Conclusion

The artificial exposure of proteinaceous paint layers to fire-related effects was examined using a combination of different vibrational spectroscopies, pyrolysis (py)-GC–MS and colourimetry. Two sets of proteinaceous model samples such as egg yolk adhesive and lead white tempera were mounted on a specially designed CH test chamber and on top of a cone calorimeter stack (CH stack). Untreated spruce was selected for the burning material. The paint layers exposed on top of the CH stack were subjected to fire effluents as well as to very high temperatures (max. ~ 450 °C), while the paint layers in the CH test chamber were affected by fire effluents and at relatively low temperatures (max. ~ 43 °C).

Differences in the molecular changes, which were observed between different batches of the exposed paint layers of egg yolk binder (E) to fire-related effects on the CH stack, probably occurred due to exposure to different absolute temperatures. Namely, the exposure to lower absolute temperatures (batch 1) favours the preservation of the most stable amino acids. Invasive and non-invasive FTIR spectra collected on the longest-exposed E model samples revealed the presence of tyrosine (TYR) amino acid. TYR amino acid has the highest melting point (T_m_ =  ~ 316 °C) among the amino acids and it was, along with other aromatic amino acids (such as tryptophan and phenylalanine), confirmed by means of the pyrolysis-GC–MS. Furthermore, the oxidation reactions of the triglycerides and the conformational changes of the proteins predominated in the longest-exposed E model samples (in all the batches) to fire-related effects. The main differences observed using the py-GC–MS of the exposed E samples in comparison to the reference (unexposed) E samples were the lower concentrations of lipid and carbohydrate pyrolysis reaction products, meaning that the exposed samples underwent polymerisation. The polymerisation effect and the crosslinking reactions were more pronounced for the batches exposed to higher absolute temperatures. A similar trend was observed with the colourimetry, where darkening and yellowing were the main differences in the colour change of the E model samples.

In the case of the lead white tempera (E + LW) exposure to fire-related effects on top of the CH stack, all the investigated batches showed comparable results. Sample dehydration with partial pigment dissociation was observed in the longest-exposed E + LW model samples on the CH stack. Namely, lead carbonate and lead oxide (a degradation product of the lead white pigment) were detected by means of vibrational spectroscopies. Moreover, lead carboxylates (soaps), which are also degradation products of lead white paints, were detected on the E + LW samples with longer times of exposure. During the py-GC–MS of the exposed E + LW, the lead white pigment caused the formation of oxidative radicals due to the lattice oxygen present in the metal oxides. This caused the oxidized and lower-molecular-weight reaction products. The carbohydrates transformed into such low-molecular-weight pyrolysis fragments that they were undetectable by GC–MS. Furthermore, the main difference causing the total colour change of the E + LW samples was related to darkening. No significant differences between the batches were observed in the colourimetric measurements.

The investigation of the proteinaceous model samples (E and E + LW) exposed to fire effluents in the CH test chamber did not show any specific changes at the molecular level. Furthermore, the total colour change of these samples was not as high as that of the samples placed on the CH stack, but still in general visible to the naked eye. A comparison of the results collected from the proteinaceous paint layers exposed to fire-related effects on the CH test chamber and CH stack shows that an increased temperature has a greater effect on the degradation of paint layers than the influence of the fire effluents.

This study turned out to be very useful for understanding some of the degradation effects of fire that might occur in museums or galleries. For the first time some selected paint materials were investigated in such conditions, and the results can be very helpful for planning successful conservation and restoration treatments of egg yolk based paint films damaged by fire. However, other types of binding media and pigments need to be investigated in order to obtain a broader database of fire-related degradation, which will be the focus of our future studies.

## Supplementary information

Supplementary Information

## Data Availability

Research data are not shared.
