# Supplementary material for: Investigation of proteinaceous paint layers, composed of egg yolk and lead white, exposed to fire-related effects
Source: Sci Rep. 2020 Nov 3;10:18961. doi: 10.1038/s41598-020-75876-y (PMC7641158; doi:10.1038/s41598-020-75876-y)
Supplement: Supplementary file 1 — Supplementary Information [file 41598_2020_75876_MOESM1_ESM.pdf]

Supplementary Information:

Investigation of proteinaceous paint layers, composed of egg yolk and lead white, exposed to fire-related effects

Lea Legan[1,*], Klara Retko[1], Kelly Peeters[2,3], Friderik Knez[4], Polonca Ropret[1,5]

[1]Research Institute, Conservation Centre, Institute for the Protection of the Cultural Heritage of Slovenia, Poljanska 40, 1000 Ljubljana, Slovenia,
[2]InnoRenew CoE, Livade 6, 6310 Izola, Slovenia
[3]University of Primorska, Andrej Marušič Institute, Muzejski trg 2, 6000 Koper, Slovenia
[4]Slovenian National Building and Civil Engineering Institute, Dimičeva 12, 1000 Ljubljana, Slovenia
[5]Museum Conservation Institute, Smithsonian Institution, 4210 Silver Hill Rd., Suitland, MD 20746

E-mail addresses: lea.legan@zvkds.si (Lea Legan), klara.retko@zvkds.si (Klara Retko), kelly.peeters@innorenew.eu (Kelly Peeters), friderik.knez@zag.si (Friderik Knez), polona.ropret@zvkds.si (Polonca Ropret)

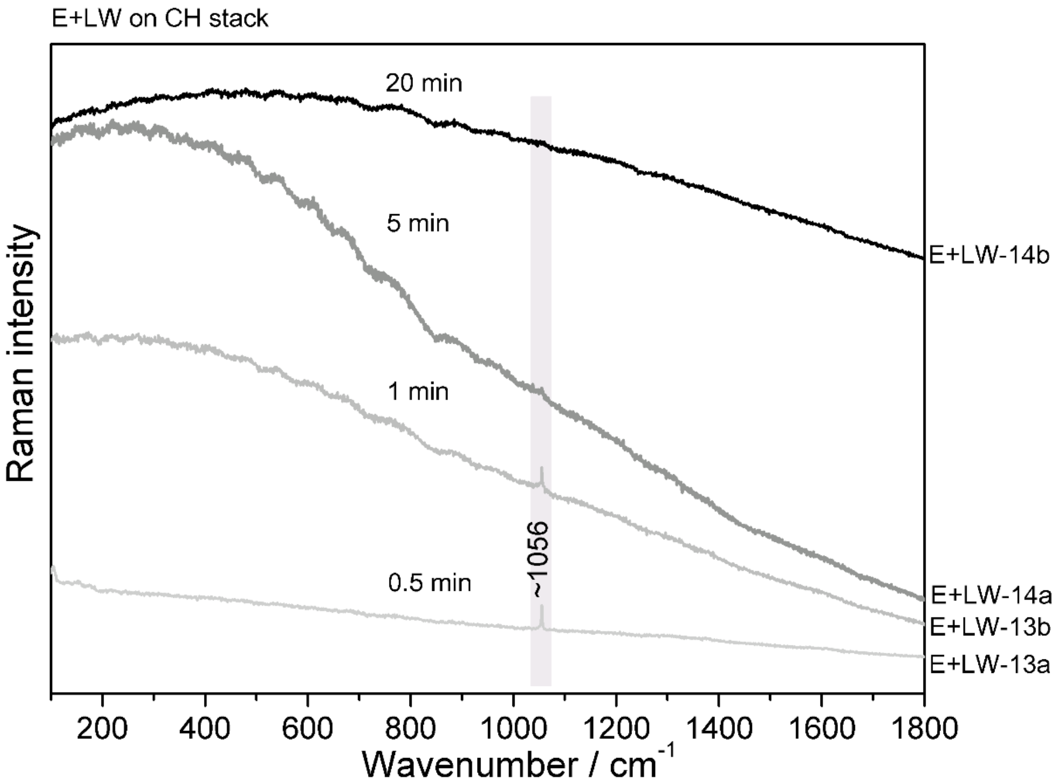

Figure S1: Raman spectra of samples E+LW exposed to fire-related effects for different times (20 min – 14b; 5 min – 14a; 1 min – 13b; 30 sec – 13a; 0 sec – 1) on CH stack. (λ_0=785 nm, ~ 3 mW, 10 s exposure, 2 accumulations).

Table S1: Comparison of pyrolysis reaction compounds of egg yolk (E-1) and burned egg yolk (E-3a and E-19b).

RT	compound	Peak height (counts)		
		E - 1	E – 3a	E – 19b
2.00	CO_2, trimethylamine	1296053		379359
2.60	1-hexene	135101	122134	105771
3.16	oxazole	158600	105189	
3.26	1-heptene	182320	120179	66301
3.67	N,N-dimethylaminoethanol	363228	68482	33824
3.95	Chloro-N,N-dimethyl ethanamine	188982		
4.16	toluene	233276	223257	160620
4.42	1-octene	84762	66011	72026
4.54	octane	30859	15126	35772
4.91	octadiene	68767	53652	30500
5.91	1-nonene	163157	112281	115244
5.97	styrene	65572	55523	219948
6.45	nonadiene	80985	62731	45102
6.58	Methyl butanoic acid	209038		
7.22	Hexanoic acid			30048
7.30	phenol	81783	241557	61355
7.42	1H-pyrrole-dione	145466	60087	78145
7.50	1-decene	293343	228551	186000
8.04	decadiene	68175	52931	43965
8.40	benzeneacetaldehyde	91717	76533	35739
8.50	methyl phenol	20860	233039	24649
8.60	n-butylbenzene	70183	46426	39743
8.80	cresol	548159	675898	282900
9.07	undecene	389376	290202	237605
9.11	methoxy phenol	200221		
9.19	undecane	26920	22867	32077
9.27	butyl-cyclohexanone	80035	36399	89566
9.58	undecadien	297069	191350	171221
9.87	Benzyl nitrile	182812	59696	76888
9.94	Dimethyl phenol	337483		
10.01	pentylcyclohexadiene	137385	60543	40320
10.46	undecatriene	93966	53520	40874
10.56	1-dodecene	393263	349696	227051
10.65	creosol	49204	418413	45163
10.67	glycerol	259821		
10.94	Dihydroxy benzofuran	102521	146399	52814
11.04	dodecadiene	376025	238618	213329
11.28	ethylphenol	187697		
11.54	dodecadiene	165177	111044	62409
11.89	trideceneacetate	69969	146709	45637
11.96	1-tridecene	321929	257431	173257
12.06	tridecane	91325	59392	60528
12.11	indole	311535	236551	104831
12.35	ethenyl-methoxy phenol		294137	
13.13	tridecadiene	97693	46859	36192
13.28	1-tetradecene	668743	489317	400363
13.35	methyl indole	371737	152878	81987
14.52	glucopyranose		1251868	
14.52	1-pentadecene	414596	Co-elution	145312
14.61	pentadecane	295699	Co-elution	462357
15.51	hexadecadiene	191540	126242	94568
15.71	cetene	258233	311522	136800
15.79	hexadecane		156938	

15.83	diethylphtalate	104009	116708	193000
16.61	tetradecadiene	249113	126031	115527
16.83	1-heptadecene	273074	248117	342926
16.89	heptadecane	153127	259850	215622
17.09	pentadecanal	30363	158796	89378
19.02	hexadecanenitrile	200752	171931	156484
19.24	Hexadecanoic acid methyl ester	322990	49132	40212
20.65	Octadecanoic acid propenyl ester	1331131	1538575	1272702
20.85	Octadecadienoic acid	204320		
20.91	Octadecanoic acid methyl ester	581147		
22.21	Octadecenoic acid pentyl ester	1641209	1532434	1304151
22.41	Octadecanoic acid propenyl ester	592834	768207	438420
24.31	Glycerol palmitate	146002	630006	1228980
25.54	Dimethoxydihydroxystilbene			768023
26.44	Cholestadiene	670233	61510	580495

Table S2: Comparison of pyrolysis reaction compounds of egg yolk (E-1) and burned egg yolk (E-3a and E-19b). Extra detected compounds after derivatization of the samples with BSTFA:TMCS (99:1)

RT	compound	Peak height (counts)		
		E - 1	E – 3a	E – 19b
1.1	acetamide	21112567		
3.49	Acetic acid	263854		
4.41	Acrylic acid	229208		
6.06	Dimethylamino ethanol	1404004		
8.42	Lactic acid	1558645		
8.55	Hexanoic acid	711414		
9.39	propenoic acid	389524		
10.01	cresol	1062356		
10.44	acetin	583766		
16.23	deoxyribopyranose		512886	90598
17.19	levoglucosan		74017	297147
18.48	Myristic acid	505357		
19.38	Palmitoleic acid	490831	74968	138416
19.61	Hexadecanoic acid	1966684	2066877	1497318
19.96	allofuranose	1201377		
20.21	Palmitelaidic acid	1003443		
20.38	Palmitic acid	28762385	446057	317097
21.24	Oleic acid	6056457	1005947	449162
21.41	Octadecanoic acid	1502963	448049	131226
22.13	Stearic acid	5214717	165262	32611
24.70	palmitoylglycerol	1085083	345770	149950
24.95	1-monopalmitin	2447431	239292	435253
26.05	cholesterol	624205	1314047	475549
26.19	1-monooleoylglycerol	926598	804253	
26.34	Glycerol monostearate	293265	380117	

Table S3: Comparison of pyrolysis reaction compounds of lead white tempera (E+LW-1) and burned lead white tempera (E-14b and E-32b).

RT	compound	Peak height (counts)		
		E+LW - 1	E+LW – 14b	E+LW – 32b
2.00	Carbon dioxide	8451887	7922651	9012420
2.60	1-hexene	170736	119678	170521
3.06	benzene	269390	195116	259933
3.26	1-heptene	273232	169503	229882
3.34	heptane	137739	128476	155549
3.67	Heptenyl acetate	118523	±40000	50112
4.16	toluene	145757	78177	97559
4.42	1-octene	292104	240474	325258
4.54	octane	279362	179835	191530
4.91	octadiene	103982	35521	36783
5.04	methyl butanoic acid	21798	58986	46309
5.18	methy butanoic acid	67495	34296	35490
5.91	1-nonene	361833	323956	461206
5.97	styrene	30511	present	
6.01	cyclohexanone		356793 (co-eluting peaks)	758495 (co-eluting peaks)
6.04	nonane	222479		
6.45	nonadiene	123592	51279	55287
6.97	Propyl benzene	64550	43078	41205
7.09	benzaldehyde	56569	68914	77765
7.22	Hexanoic acid	22423	99521	97395
7.30	phenol	194188	212298	298815
7.50	1-decene	662851	559873	722796
7.64	decane	370105	274020	326583
7.92	cycloheptanone	18790	217946	375945
8.04	decadiene	143531	77978	87454
8.40	benzeneacetaldehyde	223396	14937	26482
8.60	n-butylbenzene	223781	94584	94453
8.80	cresol			
9.07	1-undecene	955426	724164	927107
9.19	undecane	568246	405429	491527
9.58	undecadien	552625	295295	321948
9.87	Benzyl nitrile	?	59186	96351
10.01	pentylcyclohexadiene	129877	23148	17293
10.21	Octanoic acid		42352	39593
10.46	undecatriene	220154	46180	47925
10.56	1-dodecene	1041820	810571	1034467
10.62	naphtalene	237718	188876	227310
10.67	dodecane	705547	493604	595821
10.77	decanal	47214	55272	70780
11.04	dodecadiene	691734	368041	420837
11.54	dodecadiene	229671	125617	142390
11.60	Bicyclo-dodecadiene	161518	156028	198396
11.89	trideceneacetate	185169	104783	122248
11.96	1-tridecene	1013605	808178	1031147
12.06	tridecane	929482	672103	828405
12.11	indole	172539	54135	77083
12.99	Heptyl benzene	105180	95407	76253
13.13	tridecadiene	179496	74173	89025
13.28	1-tetradecene	1283818	1128812	1373882
13.37	tetradecane	671575	450304	531588
13.52	dodecanal	64918	78804	90617
14.05	cyclotetradecane	343773	159933	168927
14.52	1-pentadecene	1347384	1101876	1513104

14.61	pentadecane	652182	413921	530431
14.77	tridecanal	111929	125133	137288
15.30	nonylcyclohexane	216115	97358	100296
15.51	hexadecadiene	444014	201025	222620
15.58	cylohexadecane	288612	112951	123206
15.71	cetene	575260	451498	600699
15.79	hexadecane	276745	202335	233812
15.83	diethylphtalate	296652	115361	278590
15.96	tetradecanal	184755	198680	190390
16.61	tetradecadiene	660081	272904	353595
16.83	1-heptadecene	557373	430014	599501
17.09	pentadecanal	314160	280508	312324
17.98	hexadecenal	353655	168064	173169
18.17	hexadecanal	4043043	2282534	1891535
18.79	heptadecenal			
19.02	heptadecanone		2614504	3153783
19.02	hexadecanenitrile	3321153		
19.19	hexadecanal	151091	119738	135120
19.24	Hexadecanoic acid methyl ester	1929601	74092	51845
19.81	Palmitic acid vinyl ester	220480	49655	59763
19.91	octadecadienal	1819633	160117	176101
19.96	octadecenal	3769595	1263218	975103
20.17	octadecanal	1835364	903494	794968
20.73	octadecynenitrile	1984030		
20.73	Epoxytetradecenol acetate		1292887	1279929
20.77	octadecenenitrile	3120847	611390	650418
20.85	Octadecadienoic acid	676877		
20.91	Octadecanoic acid methyl ester	1317029		
20.94	nonadecanone		837413	1076062
20.96	octadecanenitrile	1418232		
21.115	Methyl stearate	1421065		
21.63	hexadecanamide	586720	121279	171770
22.21	Octadecenoic acid pentyl ester	155257		
22.48	Dimethylaminoethyl palmitate	936312		
23.18	Acetyl-amino-cyano-isopropyl-methylazulene	397253		
24.95	hentriacontanone	129540	727814	195162

Table S4: Comparison of pyrolysis reaction compounds of lead white tempera(E+LW-1) and burned lead white tempera (E-14b and E-32b). Extra detected compounds after derivatization of the samples with BSTFA:TMCS (99:1)

RT	compound	Peak height (counts)		
		E+LW - 1	E+LW – 14b	E+LW – 32b
1.1	acetamide	1454262		
6.06	Dimethylamino ethanol	1150301		
10.38	acetin	1045079		
11.40	Benzoic acid	481945		
18.48	Myristic acid	1893766		
19.45	Pentadeconoic acid	456226		
19.61	Hexadecanoic acid	1286779	291228	22633
20.21	Palmitelaidic acid	1000311		
20.42	Palmitic acid	35513630		
22.13	Stearic acid	878280		
24.70	palmitoylglycerol	719033		
24.95	1-monopalmitin	3149616		
26.05	cholesterol	866613	1190613	905977
26.19	1-monooleoylglycerol	1758213		
26.34	Glycerol monostearate	337577		
